# A cooperative optimization method for the layout of shared bicycle parking areas and delivery quantity

**DOI:** 10.1038/s41598-024-54647-z

**Published:** 2024-02-20

**Authors:** Hao Chen, Wenxian Wang, Ligang Cheng, Ping Li

**Affiliations:** 1 School of Economics and Business Administration, Yibin University, Yibin City, 644000 Sichuan China; 2 School of Rail Transportation, Wuyi University, Jiangmen City, 529020 Guangdong China

**Keywords:** Layout of shared bicycle parking area, Delivery quantity of bicycles, Collaborative optimization model, Walking distance, Improved genetic algorithm, Engineering, Mathematics and computing

## Abstract

With the popularity of shared bicycles in urban areas, more and more residents choose this fast and convenient mode of transportation for short-distance travel. By optimizing the layout of shared bicycle parking areas and delivery quantity, the investment cost of shared bicycle enterprises can be effectively reduced, and the convenience of residents' travel can be improved at the same time. In this paper, we develop a collaborative optimization model for the layout of the shared bicycle parking area and delivery quantity, aiming at minimizing the walking distance of residents and the investment cost of enterprises, while considering the constraints of the parking area's attractive range and the number of bicycles placed. Aiming at the characteristics of this mixed integer nonlinear problem, an improved genetic algorithm incorporating symmetric individual precision control mechanism is designed. Finally, taking the planned area between the Second Ring Road and the Third Ring Road in the northern part of Jin-niu District, Chengdu as the background, the proposed collaborative optimization model for the layout of shared bicycle parking areas and delivery quantity is applied to a real scene. The results show that after optimization, the number of parking areas is reduced by 2, and the total investment cost is reduced by about 12.2%.

## Introduction

Bike-sharing is a car rental model based on the premise that enterprises put in a large number of bicycles, and users can only use them after paying a certain deposit and rent. It can not only relieve the pressure of urban traffic, reduce traffic congestion, but also play a role in energy saving and emission reduction. In 1965, Amsterdam, the Netherlands, put some white-painted, unlocked bicycles in public areas for free use, which is generally considered to be the earliest origin of the public bicycle system in the world. But it was not until the mid-1990s that a new generation of shared bicycle projects reappeared. People rented bicycles with magnetic cards, and then this model gradually became popular all over the world^1^. At present, 535 cities in at least 49 countries around the world have established public bicycle systems. China began to introduce the public bicycle model in 2007. The government led the establishment of bicycles with piles. In 2014, it began to introduce shared bicycles without piles. The development of shared bicycles reached its climax in 2016, all kinds of shared bicycles continued to appear on the streets of major cities, becoming a hot industry sought after by investors. As far as the current situation is concerned, dockless bicycles have become the main shared bicycle model in China due to their convenience, quickness, and random access^2–4^.

However, as the use of shared bicycles becomes more and more widespread, existing problems are becoming more and more prominent: First, the supporting planning of shared bicycles have not been followed up in time. In order to seize the market, the number of shared bicycles has increased sharply, leading to the problem of coordinating urban space resources and urban traffic management has become more and more acute. Secondly, shared bicycles are parked at will to occupy public space, and it is very common for users to park at will to occupy public areas such as sidewalks and bus stops. Since shared bicycle users only have the right to use it, it is difficult to strictly enforce the law on their violations.The large number of shared bicycles requires most law enforcement teams to be deployed for management, consuming a lot of manpower and material resources. Therefore, reasonable planning of shared bicycle parking, optimizing the layout of urban shared bicycle parking areas and the amount of bicycles, and enabling residents to conveniently access and park shared bicycles can effectively reduce the impact of shared bicycles on public transportation and space, and further bring its convenient and environmentally friendly transportation characteristics into play.

The objective of this paper is to use a mathematical optimization method to give the layout of shared bicycle parking areas and the amount of bicycles in each zone in a planned area of a city, so that the investment cost of bicycle sharing enterprises can be minimized while meeting the travel requirements of residents. We apply the method to a practical case study, and the results show that the method is applicable to the layout planning of shared bicycle parking areas. The main contributions of this paper are as follows.

1. The research in this paper takes a step forward in the study of the problems related to the optimization of the layout of shared bicycle parking areas. The method optimizes the parking areas and the amount of bicycle placement from multiple perspectives, such as user travel demand, travel convenience and enterprise investment cost.
2. The mathematical planning model for optimizing the layout of shared bicycle parking areas and the amount of bicycle placement is established, taking the walking distance of residents when using shared bicycles, the construction and operation cost of enterprise parking areas and the cost of bicycle as the optimization objectives, considering the constraints of the coverage of shared bicycle parking areas and the travel demand of residents.
3. An improved symmetric genetic algorithm incorporating a symmetric individual precision control mechanism is proposed to solve the objective conflict problem of integrated optimization by using a transformed objective function approach, extending the applicability of the proposed optimization method to large-scale problems.
4. The effectiveness of the algorithm in generating the results of the shared bicycle parking area layout is demonstrated through experiments on actual arithmetic cases in the planning area of Jin-niu District, Chengdu City. The superiority of the optimization method is illustrated by comparing with the traditional method.

The rest of the paper is organized as follows: Section "Literature review" provides an overview of the research on the problem of optimizing the layout of urban shared bike parking areas. Section "Problem description" formally describes the problem. A mathematical optimization model for this problem is proposed in Section "Model formulation". In Section "Solution algorithms", an improved genetic algorithm is designed for this optimization model. Part 6 validates the model using an area in Jin-niu District, Chengdu City as a real case. The conclusions are summarized in Section "Conclusions".

## Literature review

The problem of the layout of urban shared bike parking areas is essentially a Location Problem (LP), which is generally defined as an NP problem in academic circles. However, the bicycle parking area layout problem has new characteristics based on the traditional location problem, and is more complex.

For the study of shared bicycle, the existing research directions focus on safety regulations, user preferences, and usage environment on the one hand. On the other hand, they focus on business models, development problems and suggestions for countermeasures, and urban management. De Maio P and Shaheen S studied the historical evolution of public bicycles and introduced the development of docked public bicycles^5,6^. Martens K and Washington S described how public bicycles are developed from the perspective of development policies^7,8^. Aultman-Hall L and Nelson AC et al. studied public bicycles for three aspects: safety regulations, user preferences, and usage environment^9,10^.

Meanwhile, the research objects in Europe and the United States and other countries mainly focus on docked public bicycles, so there are fewer studies on the siting of shared bicycle parking points. Sayarshad H et al. studied the initial allocation quantity decision problem of public bicycle parking points by means of a bicycle deployment (SBD) model and designing an optimization formula for public bicycle expansion^11^. Saharidis, under the investment budget constraint, developed a Saharidis established a multi-time optimization model considering three influencing factors of parking point location, vehicle allocation and parking stake quantity allocation under the investment budget constraint with the objectives of minimizing user walking time and minimizing unmet parking and pickup demand^12^. Guan L.C. established a bike-sharing parking point multi-objective location model with the objective function of minimizing construction cost and shortest total driving distance in order to solve a series of problems caused by irregular cycling of bike-sharing point multi-objective location model^13^. On the basis of using the Logit demand forecasting model to grasp user demand information, Mete S uses the split allocation model to optimize the location of the docking station in the public shared bicycle system, thereby increasing the economy and sustainability of the transportation system^14^. Maria used a combination of questionnaires and field investigations to optimize the layout of shared bicycle parking areas in Nitra City to solve the problem of urban traffic congestion^15^. Ioannis P proposed a multi-criteria analysis-based approach from a microscopic perspective that considers a number of influencing factors including geometric characteristics of the road network, parking availability, built environment, and public acceptance for planning bicycle lane and parking site layouts^16^. DogusGuler and Tahsin Yomralioglu proposed a method that combines GIS and MCDM methods in a workflow to determine the location of bike-share system stations and bike lanes^17^. Juan Carlos et al. propose a GIS-based method to calculate the spatial distribution of potential demand for trips, use a location assignment model to locate parking spot locations, and determine spatial characteristics such as parking spot capacity and the number of initial vehicle configurations^18^.

The research on dockless bicycle sharing in China is more oriented to the problem of the layout of shared bicycle parking area, and the main research methods include exploring the spatial layout using geographic information system (GIS), data mining techniques, clustering method and modeling method. The modeling method is mainly divided into two categories, the first category is a single-objective model considering only users or operators, and the objective function is mainly the shortest traveling distance for users, minimizing investment and construction cost, etc.; the second category is a multi-objective optimization model based on different objective games. The second category is multi-objective optimization model based on different objective games. The existing site-selection models mostly focus on the user's walking distance, operator's cost and user demand prediction, without considering the whole process of finding, biking and walking to the destination for users with cycling demand. The model solution mainly uses cluster analysis method, genetic algorithm, NSGA-II algorithm, reachability analysis, and hierarchical analysis method. In the research on the layout of shared bicycle parking areas, the following literatures mainly focus on optimizing the location and quantity of bicycle placement points from the perspectives of the government and operators. Jincheng Ye established a shared bicycle drop-off point demand prediction model and a drop-off point siting model, and designed an ant colony algorithm to solve the bicycle drop-off point siting model^19^. Based on the joint coverage theory, Jiawen Liu et al. established a multi-time and multi-objective optimization model considering the layout planning of shared bicycle parking points and the initial vehicle allocation decision of each parking point^20^. He Liu established a two-tier planning model from the perspective of residents' travel demand and transportation facility supply^21^. Yunzhong Cao optimized the layout of bike-sharing parking points in different neighborhoods from the perspective of urban space management, taking the urban road access rate and bicycle usage specification as the research objectives^22^. Shuang Jin established a two-level planning traffic allocation model with user travel cost minimization and bike-sharing usage maximization as the upper-level planning and different feeder options for travelers and different transportation stops as the lower-level planning, and implemented genetic algorithm to solve the model with MATLAB programming^23^. Lingsu Wang analyzed the travel characteristics of shared bicycles in four aspects: time characteristics, distance characteristics, spatial characteristics, and cycling road segment preferences, proposed a GCN-LSTM shared bicycle short-time traffic demand prediction model with deep learning theory, and established a shared bicycle recommended parking site selection model based on the cost of shared bicycle enterprises and user satisfaction, and solved the model using NSGA-II algorithm^24^. Yujing Fu developed a two-layer planning model that simultaneously considers the bike-sharing site location problem and the section selection problem with physically separated bicycle lanes. The upper-level model aims at minimizing the total cost, including travel time cost and infrastructure construction cost; the lower-level model extends the multi-mode selection model and the path selection model, and employs a super-heuristic approach to solve the model^25^. Xuanyang Qin made a study on the scale and layout of shared bicycle feeder facilities for residential rail transit stations in old cities. In this study, a traveler benefit calculation model based on LODSUM differences, a shared bicycle travel demand prediction model based on the growth coefficient method, a shared bicycle parking site pre-selection model with the objective of shortest total walking distance, and a MOORA multi-objective optimal site selection model were developed and solved using a genetic algorithm^26^. Taking Xicheng District of Beijing as an example, Jiangyan Gu conducted a spatio-temporal analysis of shared bicycles, established an electric fence site selection model based on the maximum coverage problem, and used simulated annealing algorithm to solve the model^27^. Linfeng Li used the analysis function and statistical function of ArcGIS to obtain a site-suitability grading map, and compared and corrected the results with the clustering results of demand points to obtain an optimized siting plan for shared bicycle virtual parking spots^28^. Yiming Li researched the siting of shared bicycle drop-off points considering user demand during the morning and evening peak periods. In this study, maximizing user demand was taken as the optimization objective, and a shared bicycle parking point layout optimization model was established, and the model was solved using a clustering algorithm^29^. The following literatures consider the influence of travelers' walking distance in the study of shared bicycle parking area layout. Yu-Kun Shi used the Canopy-K-means clustering algorithm for bike-sharing demand prediction. Based on a full understanding of user demand, a shared bicycle parking site location model was developed with the lowest construction cost and the shortest total travel distance as the objective functions, and a modified NSGA-II algorithm was used to solve the model and obtain a parking site location plan^30^. Fengjie Luo develops a robust site-selection model based on travel demand uncertainty under the constraints of cost and minimization for enterprises and individuals for optimizing the layout of bike-sharing parking spots^31^. Xiaoyang Zhu established a mixed-integer linear planning siting model with ground rent cost and user walking distance cost as the objective function and the number of psychologically safe empty spaces for users as the constraint to realize the study of single-vehicle parking spot siting^32^. Yantao Liu established a borrowed parking demand prediction model based on random utility theory, and established a parking point planning layout model with the optimization objective of minimizing the sum of users' walking distance for car seeking in the planning area, and solved it with an improved genetic algorithm^33^. Qian Hou targeted the time from the departure point until the user finds the bike-sharing bike, and based on the 0–1 variable model and multi-objective optimization model for the research and layout optimization of shared bicycle parking points^34^. However, the above researches don’t consider the service area limitations of shared bicycle parking points, which often leads to unreasonable results of walking distance exceeding cycling distance.

In summary, the existing studies have achieved rich results in the layout of shared bicycle parking spots, but the above studies have optimized the layout of shared bicycle parking areas mainly from the perspective of enterprise revenue and urban land, considering input costs and the number of users, while ignoring the constraints on the service scope of shared bicycle parking areas and the impact of bicycle placement on travel demand. As mentioned earlier, these omissions can lead to unmet travel demand at certain demand points and mass idleness of shared bicycles in certain parking areas.

## Problem description

**Figure 1:**
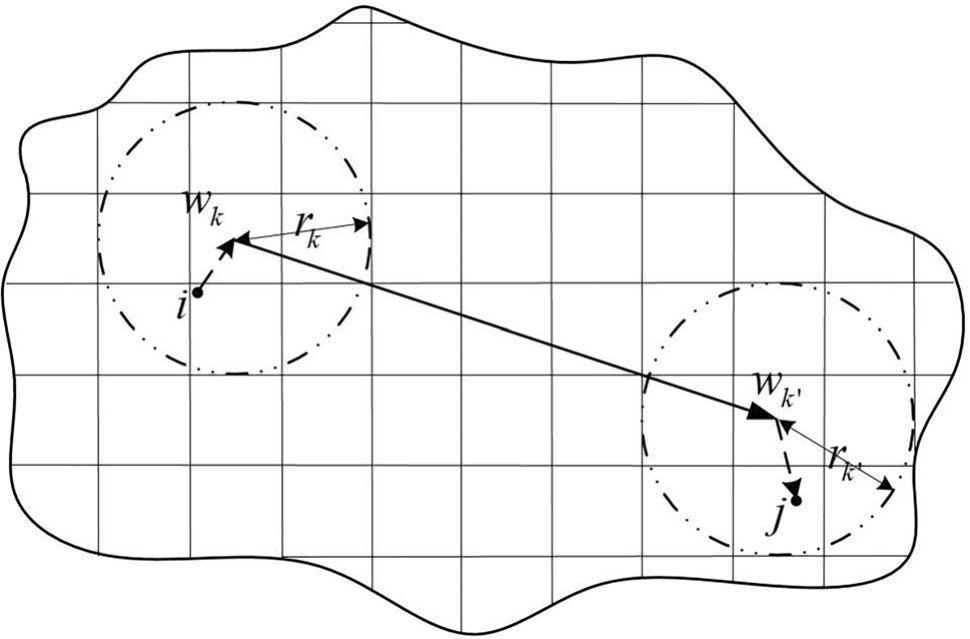
Design of basic parameters for parking areas in the region.

The nodes in the shared bicycle area system mainly include two types—travel demand points and shared bicycle parking points. Among them, the travel demand point is the main starting and ending point of shared bicycle users ' travel, and the shared bicycle parking point is a fixed area for placing shared bicycles. A schematic diagram of a simple shared bicycle regional network is $G = (U \cup W,A)$ shown in Fig. 1. Each shared bicycle parking point in the figure has a certain coverage area, which is generally a circle with an attractive radius $r_{k}$. In the process of choosing a shared bicycle from a demand point $i$ to a demand point $j$, residents need to walk to a shared bicycle parking spot $k$ that covers the travel demand point $i$, and then ride to a shared bicycle parking spot $j$ that covers the travel demand point $k^{\prime}$, get off and continue walking to the point of travel demand $j$. If the travel demand point $i$ is covered by several parking points at the same time, the passengers at the travel demand point $i$ choose to go to the nearest shared bicycle parking point.

This paper defines the problem of synergistic optimization of shared bicycle parking area layout and bicycle placement as a problem that refers to selecting a number of nodes from the existing alternative points of shared bicycle parking areas in the planning area according to certain principles and perspectives, setting them as shared bicycle parking areas and specifying the number of bicycles to be placed in each parking area; the problem mainly considers two levels: residents and shared bicycle operating companies.

### Resident level

Usually, urban residents do not choose to walk too far in the process of travel. From the perspective of facilitating residents' travel, the layout of shared bicycle parking areas will consider the following two goals: first, to meet residents' travel demand as much as possible, i.e., to build enough shared bicycle parking areas to cover the travel demand points, and to invest enough shared bicycles in each shared bicycle parking area; second, to ensure the convenience of residents. If the walking distance from the travel demand point to the bicycle parking area is too far, the meaning of bicycle sharing is not achieved. Therefore, when setting up parking areas, the extra walking distance should be shortened as much as possible.

### Enterprise level

The main consideration at the bicycle sharing operation enterprise level is economic efficiency, that is, minimizing the total cost required for the construction of the shared bicycle parking system. The shared bicycle parking area will incur a number of costs during the construction process, mainly including the construction and operation of the shared bicycle parking area and the cost of placing shared bicycles. By determining the location of the parking points, the capacity of the parking points and the distribution of the number of shared bicycles at each parking point, it is possible to meet the By determining the location of the parking sites, the capacity of the parking sites and the number of shared bicycles at each parking site, the comprehensive investment costs of the shared bicycle operating companies will be reduced as much as possible while meeting the demand of residents who choose to travel by shared bicycle.

## Model formulation

### Notation

The notations used in this paper is showed in Table 1.

**Table 1:** Notations used in the paper.

General subscripts	
$h,i,j$	Index of demand points
$k$	Index of alternative parking points
$m$	Total number of demand points
$n$	Total number of alternative parking points
Parameters and sets	
$U = \{ u_{i} \}$	A collection of demand points in the region
$W = \{ w_{k} \}$	A collection of alternative parking points in the region
$Q = \{ q_{ij} |i,j \in U\}$	Shared bicycle trips between demand points $u_{i}$ and $u_{j}$
$D_{uu} = \{ d_{ij} |i,j \in U\}$	The distance between demand point $u_{i}$ and $u_{j}$ (unit, meter)
$D_{uw} = \{ d_{ik} |i \in U,k \in W\}$	The distance between demand point $u_{i}$ and the alternative parking area $w_{k}$(unit, meter)
$q_{ij}^{ * }$	The actual number of people traveling on shared bicycles between demand points $u_{i}$ and $u_{j}$
$r_{k}$	Attractive distance length of shared bicycle parking area $w_{k}$ (unit, meter)
$\overline{l}$	The upper limit of the expected walking distance of residents during travel
$c_{k}$	The construction cost of setting up the parking point at the alternative point (unit, $k$ yuan)
$B_{k}$	The maximum amount of shared bicycle in parking point $k$
$S_{i}$	A collection of parking points covering demand points $u_{i}$
$c_{0}$	Unit price of shared bicycles (unit, yuan/vehicle)
$\theta$	Travel demand satisfaction coefficient
Variables	
$x_{k}$ as 0–1variable	Whether to set a shared bicycle parking point at alternative point $w_{k}$. If yes, the value is 1, if not, the value is 0;
$\delta_{ik}$ as 0–1 variable	Whether the demand point $u_{i}$ is covered by the parking point $w_{k}$.If yes, the value is 1, if not, the value is 0;
$y_{k}$ as an integer variable	After alternative point $w_{k}$ is set as a shared bicycle parking point, the number of shared bicycles planned to be put into this point is $y_{k}$

### Assumptions

The following assumptions are made throughout this article:

1. In this paper, demand points are used to represent a small area. The demand for using and parking bicycles in this area is concentrated in the demand points. The walking distance of residents in the demand points is negligible, and the travel demand of the demand points is also negligible;
2. Assume that the shared bicycle parking area can meet the travel demand of any demand point within its attractive range;
3. It is assumed that residents know the distribution of shared bicycle parking areas within the maximum walking range, and choose the nearest parking area according to the "proximity principle";
4. If there is no shared bicycle in the parking area that the resident goes to, they will choose other means of transportation to the destination.

### The model

Model I of the synergistic optimization model for the layout of shared bicycle parking area and delivery quantity is shown in (1) to (11).

1 $$ \min Z_{1} = \sum\limits_{i,j \in U} {\sum\limits_{k \in W} {q_{ij}^{ * } \cdot \left[ {\min \{ d_{ik} \} \delta_{i,k} + \min \{ d_{kj} \} \delta_{j,k} } \right]} } + \sum\limits_{i,j \in U} {\left( {q_{ij} - q_{ij}^{ * } } \right) \cdot d_{ij} } $$

2 $$ \min Z_{2} = \sum\limits_{k} {c_{k} x_{k} } + c_{0} \sum\limits_{k} {y_{k} } $$

Subject to

3 $$ \sum\limits_{k} {\delta_{ik} } \ge 1;\forall k \in W $$

4 $$ x_{k} \ge \delta_{i,k} ;\forall i \in U,k \in W $$

5 $$ J(r_{k} - d_{ik} ) \ge x_{k} \cdot \delta_{ik} ;\forall i \in U,k \in W $$

6 $$ \sum\limits_{k} {x_{k} \delta_{ik} y_{k} } + \sum\limits_{h} {q_{hi} } \ge q_{ij} \cdot \theta ;\forall k \in W,\forall h,i,j \in U,h \ne i,j $$

7 $$ q_{ij}^{ * } \le q_{ij} ;\forall i,j \in U $$

8 $$ \min \{ d_{ik} \} \delta_{i,k} + \min \{ d_{kj} \} \delta_{j,k} \le \overline{l};\forall i,j \in U,k \in W $$

9 $$ y_{k} \le B_{k} \cdot x_{k} ;\forall k \in W $$

10 $$ x_{k} ,\delta_{i,k} \in \left\{ {0,1} \right\};\forall i \in U,k \in W $$

11 $$ y_{k} \in Z;\forall k \in W $$

The objective function (1) minimizes the total walking distance for all residents, and the objective function (2) minimizes the total cost of building and operating of placing the shared bicycle. Constraint (3) requires all demand points to be covered by at least one and more parking areas. Constraint (4) ensures that the parking area coverage is based on the premise that the parking area is set up at that alternative point, i.e., the range where the parking area is acceptable to residents when they choose to travel by shared bicycle. Constraint (5) indicates that the demand point is covered by the parking area provided that the distance between the two is less than the distance covered by the parking area. Constraints (6) to (7) indicate that at least a certain percentage of travel demand must be satisfied. Constraint (8) is the upper limit of the total walking distance for residents in the process of using shared bicycle for travel. Constraint (9) determines the upper limit of bicycle placement in the parking area. Constraints (10) to (11) are decision variable constraints.

## Solution algorithms

The optimization model of shared bicycle parking area layout considering multiple constraints suffers from the dimensionality problem due to the rapidly increasing number of Eqs. (1) to (11). Therefore, a widely used heuristic algorithm, namely genetic algorithm (GA), is used to obtain satisfactory solutions faster in solving models with more nodes and larger scale.

Genetic algorithm is a randomized search method that simulates Darwin's idea of "survival of the fittest" and evolves from the laws of evolution such as survival of the fittest and survival of the fittest in the biological world. The algorithm borrows several concepts from biogenetics (such as chromosome, gene, population, replication, mating, variation, parent, offspring, adaptation, etc.), and simulates natural selection and biological genetic process. It is often used for solving large-scale combinatorial optimization problems.

There is a contradictory relationship between the two objective functions in Model I, Eq. (1) wants to have more parking points the better, while Eq. (2) wants to have less the better. Using the basic genetic algorithm to solve this multi-objective optimization problem leads to the following problems.

1. The multi-objective genetic algorithm has poor local search ability, and after a certain number of generations of genetic inheritance, the new individuals resulting from close individual similarity are highly similar, thus failing to produce new individuals.
2. Slow convergence of the optimal solution, or even difficulty in reaching the region of the optimal solution.
3. Reduced operation speed due to high parameter complexity.

Therefore, this paper introduces the fusion symmetric individual precision control mechanism to improve the design of the basic genetic algorithm's encoding method, selection method and other operators.

### Solution space structure

When genetic algorithm is used to solve an optimization problem, the solution code of the problem is first defined as a natural array with certain design rules. In this paper, a 0–1 vector is used to represent the layout scheme of the parking area, and the initial chromosome $chrom$ is designed.

$$ chrom = [x_{1} ,x_{2} , \cdots ,x_{i} , \cdots x_{n} ] $$

where each gene locus $x_{i}$ in the chromosome takes the value $\{ 0,1\}$, for whether the stopping area is set for that alternative point, with 1 being yes and 0 being no.In order to solve the problem that the genetic algorithm iterations lead to the similarity of individuals, but it can't produce new individuals. The design of random placement of special individuals, called "symmetric individuals" in this paper. The individuals between symmetric individuals $C$ and $C^{ * }$ are satisfied with $x_{i} = 1 - x_{i}^{ * }$. The advantage of randomly generating "symmetric individuals" is that it generates a richer population diversity, and at the same time serves to continuously open up space when genetic segments are not crossed and mutated. Symmetric individuals are placed when the variation between the mean fitness value and the best fitness value of a contemporary population is controlled within a certain precision or when the variation between the best fitness values of two adjacent generations is within a certain control precision. The control precision can be either relative or absolute control precision.

### Fitness function

For the two objective functions in the model, this paper combines the weighting method to re-determine the objective function value^20^. At the same time, in the actual situation of urban roads, some roads are often disconnected. In order to express such road conditions, a fitness function, namely a penalty function, is introduced. The individual fitness is mainly determined by the objective function value. In this paper, when calculating the chromosome fitness value, for some gene values that exceed the limit or cannot be realized, a penalty coefficient $+ \infty$ and a step function $J(x)$ are introduced, which are defined as:

12 $$J(x) = \left\{ {\begin{array}{*{20}c}    {1,\,} & {L_{{ij}}  =  + \infty \,i \ne j}  \\    {0,\,} & {else}  \\   \end{array} } \right.{\text{ }}$$

Incorporate the penalty function into the objective function,

13 $$ F = M \cdot J(x) + \omega_{1} \cdot Z_{1} + \omega_{2} \cdot Z_{2} $$

### Genetic operators

#### Options

In this paper, we adopt the "elite retention" selection strategy, which requires the optimal setting of parking points according to different initial schemes, and the relatively better individuals directly enter the offspring, and then perform random traversal sampling of the current population until the population size of the offspring is the same as that of the parent.

The control precision $\varepsilon_{1} ,\varepsilon_{2}$ and symmetry factor $\lambda$ of the input symmetric individuals are introduced, and the symmetric individuals are placed after the amount of change between the average adaptation value and the best adaptation value of individuals in a certain generation or the amount of change in the best adaptation value of two adjacent generations reaches a certain control precision, and $2\left\lfloor {\lambda \times M} \right\rfloor$ symmetric individuals are randomly generated for placement. The symmetrical individuals placed are set as seed individuals, and the individuals generated directly through roulette are non-seed individuals. When performing the crossover operation, the seed individual can randomly select an individual in the non-seed group to perform the operation with it, and the remaining non-seed individuals perform pair wise operation.

#### Crossover and mutation

**Figure 2:**
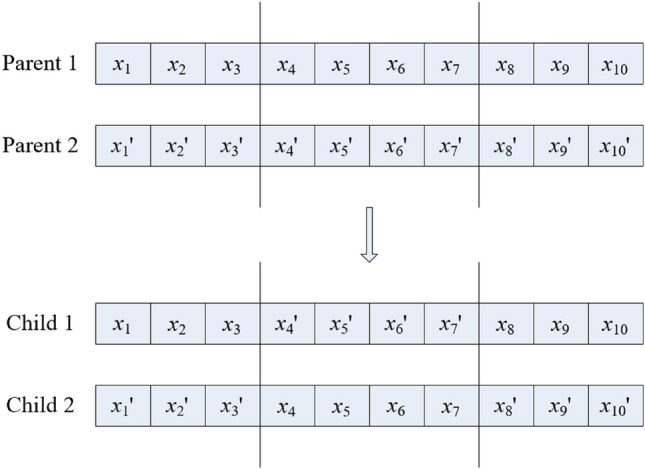
Schematic diagram of crossover operation.

The crossover operation in the genetic algorithm is improved to take the area crossover operation, as shown in Fig. 2. At the same time, the mutation operation is improved to randomly select a gene locus in an individual for mutation, and the mutation method is to change its value, ie $x_{i} \leftarrow 1 - x_{i}$.

### GA algorithms

The steps of the improved genetic algorithm for solving the model are as follows:

*Step 1* Algorithm initialization: set the algebraic counter, the initial algebra $n \leftarrow 1$, set the maximum evolutionary algebra $T$, and randomly generate populations, symmetry factor is $\lambda$, and cast symmetrical individual control accuracy $\varepsilon_{1} ,\varepsilon_{2}$, and form a parent group.

*Step 2* Individual evaluation: Calculate the objective function of the individual in the initial parent group, that is, the fitness value.

*Step 3* Selection operation: apply the selection operator to the parentgroup. The purpose of selection is to directly inherit the optimized individuals to the next generation or generate new individuals through paired crossover and then inherit them to the next generation. The selection operation is based on the fitness evaluation of the individuals in the population.

*Step 4* Input of symmetrical individuals: If the variation between the average fitness value and the best fitness value of a certain generation of individuals is within the precision $\varepsilon_{1}$, or the variation of the best fitness value of two adjacent generations is within the precision $\varepsilon_{2}$, the roulette method is used to generate $M - 2\left\lfloor {\lambda \times M} \right\rfloor - 1$ number of new generation Individuals in the group, and $2\left\lfloor {\lambda \times M} \right\rfloor$ number of symmetric individuals are randomly generated, denoted as $C_{1} ,C_{2} , \cdots ,C_{{2\left\lfloor {\lambda \times M} \right\rfloor }}$, and added to the next generation. At this time, the group composed of symmetric individuals is called for the seed subgroup, and the group composed of other new generation individuals directly generated by roulette is called for the non-seed population.

*Step 5* Crossover operation: applying the crossover operator to the population. The central role in the genetic algorithm is played by the crossover operator, which is used to generate offspring individuals. The crossover operator is applied to the seeded individuals in Step4, and one of the non-seeded individuals can be randomly selected to perform the operation with them, and the remaining non-seeded individuals are then paired in pairs.

*Step 6* Mutation operation: apply the mutation operator to the population. That is to change the gene values at some loci of individual strings in the population to generate new offspring. Population algebra $P(t)$ obtains the next generation population $P(t + 1)$ after selection, crossover and mutation operations. If the best adaptation value of $M$ individuals generated above is less than the best adaptation value of the previous generation, then the best individual of the previous generation replaces the worst individual of the current generation.

*Step 7* Termination condition judgment: when the genetic algebra $n$ is the maximum evolutionary algebra, immediately, $n = N$, the individual with the maximum fitness obtained in the evolution process is output as the optimal solution, and the calculation is terminated. At this time, the layout scheme of the shared bicycle parking area can be considered as the optimal solution obtained.

## Numerical experiments

### Basic experiments for an idealized district

**Figure 3:**
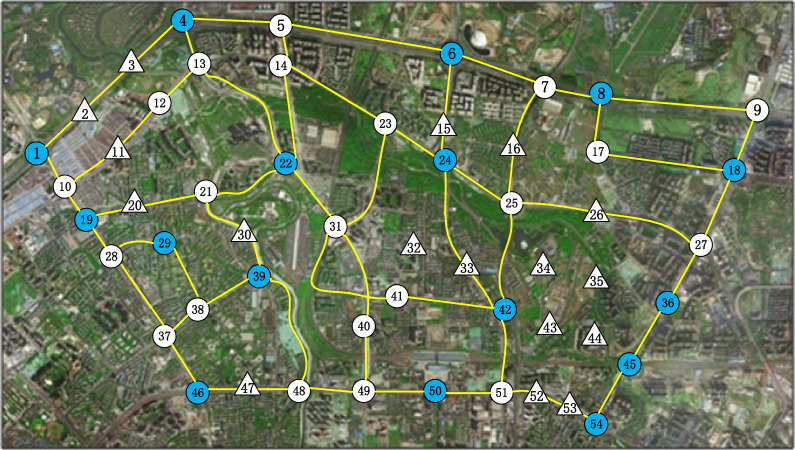
Schematic diagram of the roads in the planning area of shared bicycles in Chengdu.

This section describes the layout planning area of the shared bicycle parking area between the North Second Ring Road and the Third Ring Road in Jinniu District, Chengdu City, as shown in Fig. 2. The transportation layout data processing method in reference^35^ was used to process the relevant GIS data, forming the corresponding topology structure as shown in Fig. 3.

**Figure 4:**
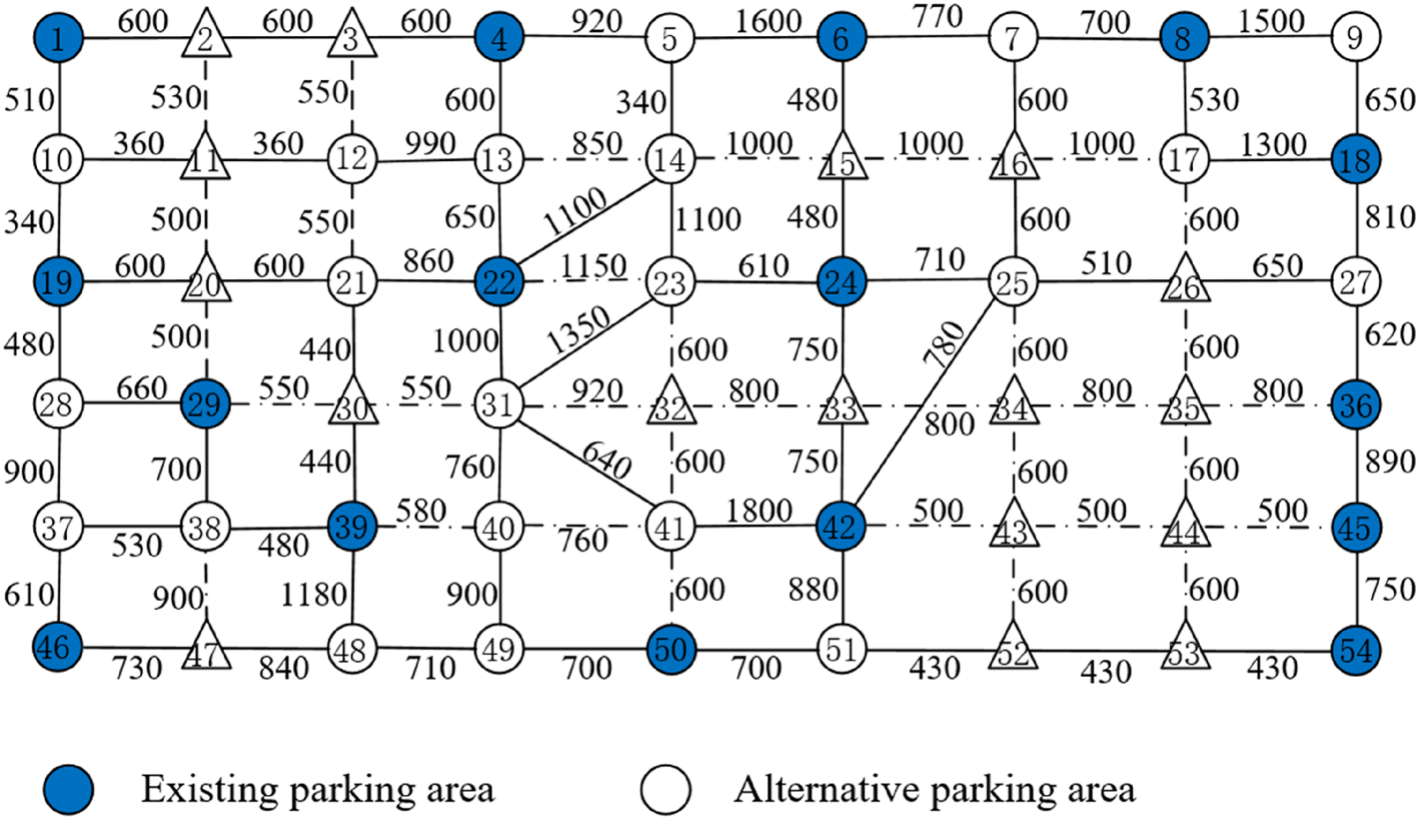
Schematic diagram of the current shared bicycle parking area.

There are 52 travel demand points in the area, of which 37 points are used as alternative shared bicycle parking areas, which is showed in Fig. 4. The numbers in Fig. 4 indicate the distance of the road section, the solid blue circles indicate the shared bicycle parking areas set in the current layout scheme, and the hollow circles indicate the alternative shared bicycle parking areas. The solid lines indicate that the road is passable, and the dashed lines indicate that the road is not suitable for shared bikes, but pedestrians are allowed to pass it on foot.

According to the travel volume data collected by Hello-Bike Sharing Company in November 2021, there are 376 travel demands with different destinations. The construction cost of setting up parking areas at each alternative point and the unit price of shared bicycles refer to the account of Hello Bike Sharing Company. Other relevant parameters are as follows: the attraction distance of the shared bicycle parking area $r_{k} = 1500\,m$, the upper limit of the walking distance of residents is set based on the results of the questionnaire survey $\overline{l} = 1500\,m$, the maximum number of bicycles in each parking area $B_{k} = 200$, and the travel demand satisfaction coefficient $\theta = 90\%$.

### Experiments for Jin-niu district in Chengdu

**Figure 5:**
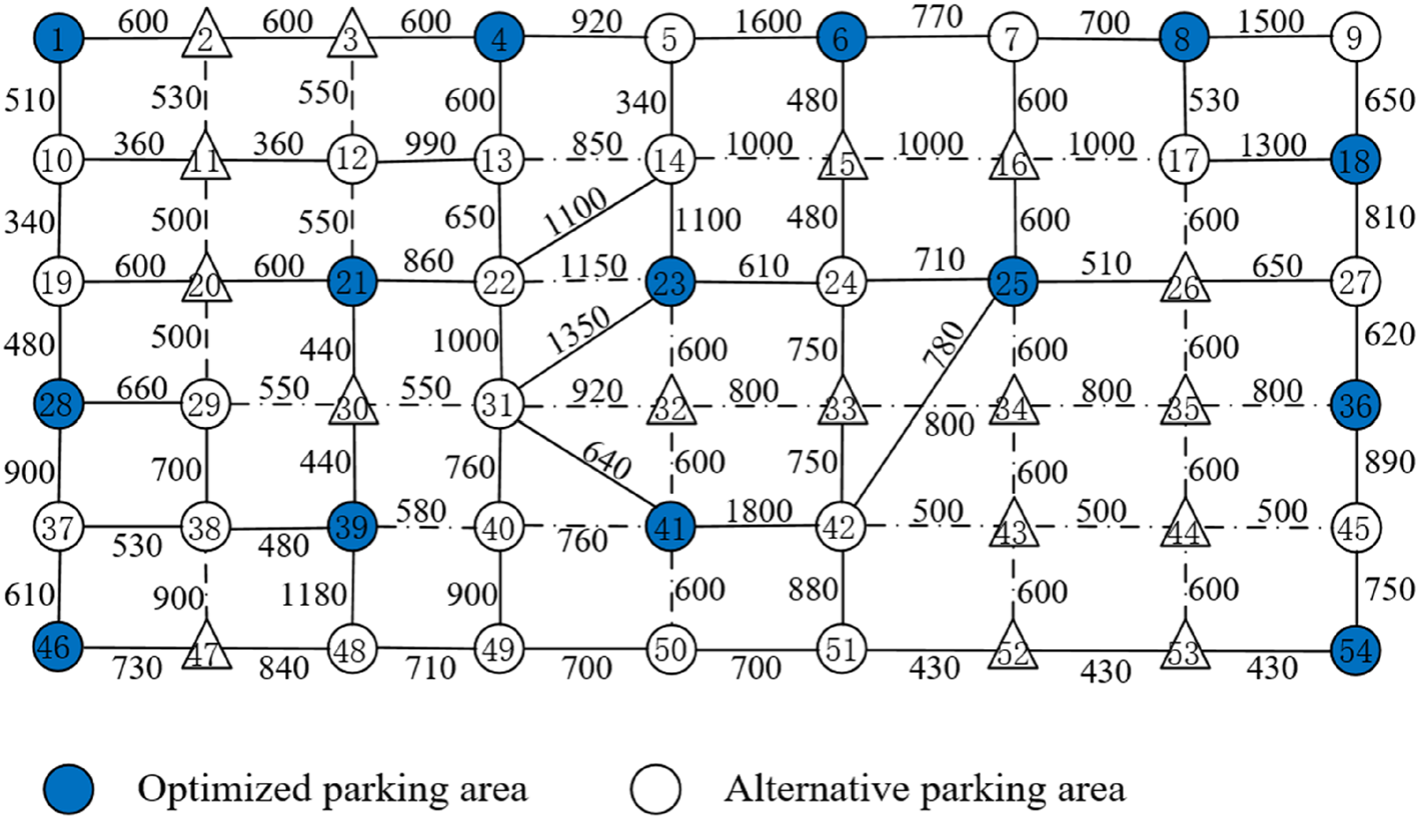
Schematic diagram of optimized shared bicycle parking area.

According to the model mentioned above, after trial calculation and adjustment, the values of each parameter of the algorithm are finally determined: maximum evolution algebra $N = 1000$, crossover probability $p_{c} = 0.75$, and mutation probability $p_{m} = 0.05$, set $\omega_{1} = \omega_{2} = 0.5$. The entire solution process is based on Matlab2012a software, and it tends to be stable after about 110 iterations. The optimal solution is finally obtained as shown in Fig. 5.

As is showed in Table 2, compared with the existing layout scheme of the shared bicycle parking area, the number of layout points required for the optimized layout scheme of the shared bicycle parking area is reduced from 16 to 14, and the number of bicycles is reduced from 1710 to 1620. The total investment cost of the enterprise decreased from 3,483,217.5 yuan to 3,059,784.7 yuan, a decrease of 12.2%. The total walking distance of residents dropped from 1,726,530 m to 1,635,960 m, a decrease of 5.24%. Table 3 shows the number of demand points covered by each parking area in the layout schemes of existing and optimized shared bicycle parking areas. In the existing layout scheme of shared bicycle parking areas, the number of demand points covered by each layout point is 4 to 13. The average distance between the demand point and the parking point is 981 m. In the optimized layout scheme of the shared bicycle parking area, the number of demand points covered by each layout point is 5to 15. The average distance between the demand point and the parking point is 1073 m. It can be found that the amount of shared bicycles and the number of coverage demand points in most parking points have increased, so that the overall cost of the shared bicycle parking area layout scheme is reduced under the premise of basically meeting the regional travel needs.

**Table 2:** Existing and optimized layout schemes of shared bicycle parking areas.

Existing layout scheme (Total:1710)				Optimized layout scheme (Total:1620)			
Layout point	Volume	Layout point	Volume	Layout point	Volume	Layout point	Volume
*x*_1_	100	*x*_29_	120	*x*_1_	100	*x*_28_	120
*x*_4_	80	*x*_36_	130	*x*_4_	80	*x*_36_	130
*x*_6_	80	*x*_39_	110	*x*_6_	80	*x*_39_	110
*x*_8_	70	*x*_42_	100	*x*_8_	70	*x*_41_	170
*x*_18_	140	*x*_45_	120	*x*_18_	150	*x*_46_	180
*x*_19_	80	*x*_46_	200	*x*_21_	120	*x*_54_	120
*x*_22_	90	*x*_50_	100	*x*_23_	110		
*x*_24_	120	*x*_54_	70	*x*_25_	80

**Table 3:** Comparison of coverage number for layout point.

Existing layout scheme				Optimized layout scheme			
Layout point	Coverage	Layout point	Coverage	Layout point	Coverage	Layout point	Coverage
*x*_1_	9	*x*_29_	13	*x*_1_	9	*x*_28_	10
*x*_4_	8	*x*_36_	7	*x*_4_	8	*x*_36_	7
*x*_6_	7	*x*_39_	12	*x*_6_	7	*x*_39_	14
*x*_8_	7	*x*_42_	12	*x*_8_	7	*x*_41_	11
*x*_18_	6	*x*_45_	8	*x*_18_	6	*x*_46_	5
*x*_19_	11	*x*_46_	4	*x*_21_	15	*x*_54_	6
*x*_22_	11	*x*_50_	9	*x*_23_	11		
*x*_24_	12	*x*_54_	6	*x*_25_	14		

**Figure 6:**
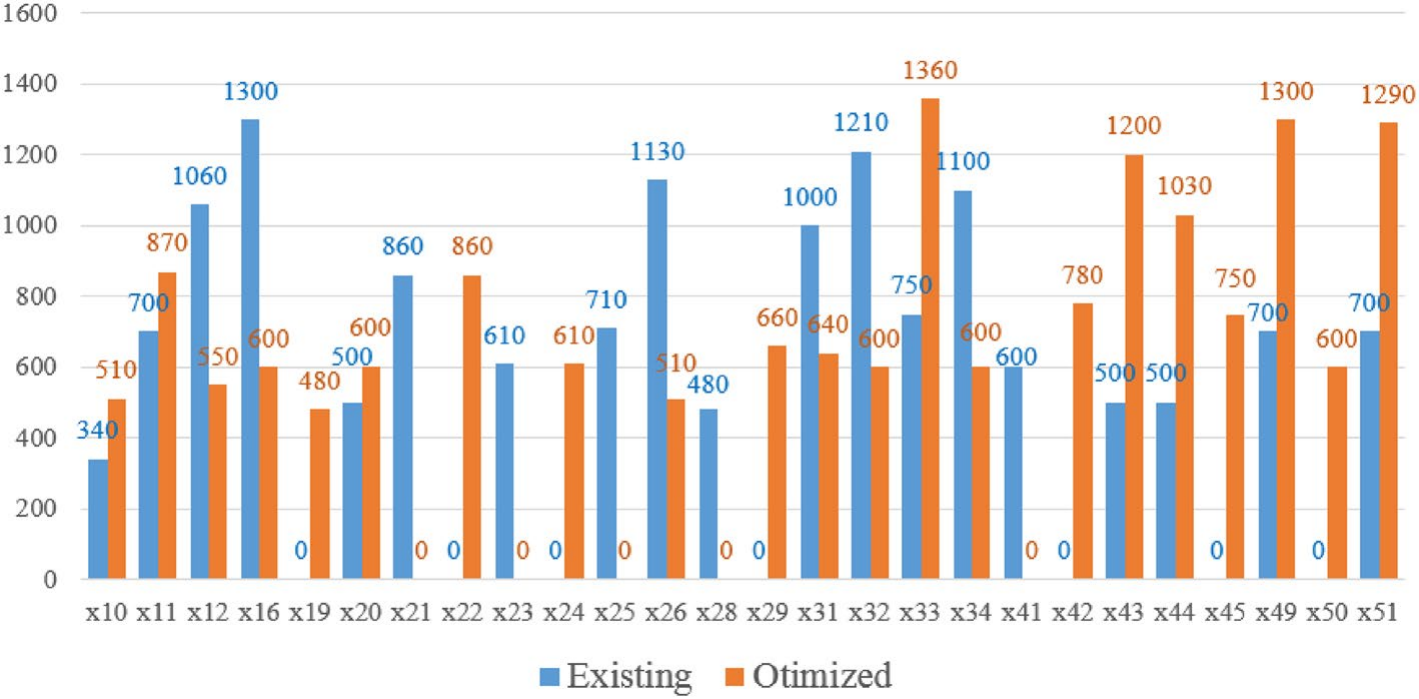
Comparison of the walking distance of each demand point.

From the shortest walking distance from the demand point to the parking area (Tables 4, 5), it can be seen that compared with the existing parking area layout scheme, the number of demand points with a walking distance of 0 in the optimized parking area layout scheme is reduced by 2, But the number of demand points with a walking distance of more than 1000 m is reduced by 1. This is because the reduction in the number of parking areas will inevitably lead to an increase in the walking distance of some demand points. The comparison between the existing scheme and optimized scheme is shown in Fig. 6.

**Table 4:** Walking distance in the existing bicycle sharing parking area layout scheme.

Demand point	Walking distance	Demand point	Walking distance	Demand point	Walking distance	Demand point	Walking distance	Demand point	Walking distance
1	0	12	1060	23	610	34	1100	45	0
2	600	13	600	24	0	35	800	46	0
3	600	14	1100	25	710	36	0	47	730
4	0	15	480	26	1130	37	610	48	1180
5	920	16	1300	27	620	38	480	49	700
6	0	17	530	28	480	39	0	50	0
7	700	18	0	29	0	40	580	51	700
8	0	19	0	30	440	41	600	52	860
9	650	20	500	31	1000	42	0	53	430
10	340	21	860	32	1210	43	500	54	0
11	700	22	0	33	750	44	500

**Table 5:** Walking distance in the optimized bicycle sharing parking area layout scheme.

Demand point	Walking distance	Demand point	Walking distance	Demand point	Walking distance	Demand point	Walking distance	Demand point	Walking distance
1	0	12	550	23	0	34	600	45	750
2	600	13	600	24	610	35	800	46	0
3	600	14	1100	25	0	36	0	47	730
4	0	15	480	26	510	37	610	48	1180
5	920	16	600	27	620	38	480	49	1300
6	0	17	530	28	0	39	0	50	600
7	700	18	0	29	660	40	580	51	1290
8	0	19	480	30	440	41	0	52	860
9	650	20	600	31	640	42	780	53	430
10	510	21	0	32	600	43	1200	54	0
11	870	22	860	33	1360	44	1030

### Algorithm validation

In order to further verify the performance of the model and algorithm, in this section we created 6 sets of simulation cases of shared bicycle parking area layout, and compared the improved genetic algorithm used in this paper with the basic algorithm. Algorithm parameter settings are the same as above. These cases are quoted in n-k-d form—for example, 20–50–500 corresponds to the case of 20 alternative parking points, 50 demand points and 500 destination trips.

Table 6 shows the comparison of the improved genetic algorithm used in this paper and the basic genetic algorithm in solving problems of different scales. We record the objective function value and CPU running time (CPU/S) when the two methods solve these instances.

**Table 6:** Performance comparison of CPLEX and the GA algorithms.

Instances	Improved Genetic Algorithm		Basic Genetic Algorithm		CPLEX	
	Walking distance	Cost of investment	Walking distance	Cost of investment	Walking distance	Cost of investment
20–50–400	1,722,979.1	3,222,539.2	1,744,861.0	3,517,079.3	1,722,979.1	3,222,539.2
20–80–600	2,506,151.5	4,687,329.8	2,564,795.4	5,055,285.1	2,506,151.5	4,687,329.8
30–100–900	3,915,861.7	7,323,952.7	4,014,541.4	7,773,643.4	3,834,657.5	7,301,884.2
50–120–1200	4,751,245.5	8,886,396.0	5,180,283.0	9,528,882.4	4,439,567.3	8,637,495.5
70–150–1600	6,683,070.6	12,499,546.0	7,197,667.1	13,659,503.9	–	–
90–200–2000	7,918,742.6	14,810,660.0	8,531,653.2	15,169,078.0	–	–

As shown in Table 6, the optimal solution obtained by applying the improved genetic algorithm to solve the model is better than the optimal solution obtained by the basic genetic algorithm , and the quality gap of these solutions is distributed between 1.27% and 9.81%. In terms of calculation time, the running efficiency of the improved genetic algorithm is higher than that of the basic genetic algorithm, but there is no significant difference between them. When the number of alternative parking points exceeds 70, CPLEX cannot obtain the optimal solution. These results validate the effectiveness of the proposed improved genetic algorithm and illustrate its superiority in finding optimal solutions when dealing with large-scale problems.

## Conclusions

In this paper, we investigate the problem of the layout of urban shared bicycle parking areas and propose a cooperative optimization method for the layout of shared bicycle parking areas and delivery quantity for daily scheduling and operation of shared bicycle enterprises. In addition, we design an improved genetic algorithm incorporating a symmetric individual precision control mechanism to solve this problem, which can obtain the optimal solution in an acceptable time. It outperforms the basic genetic algorithm in terms of computational efficiency and solution quality. The applicability of the improved genetic algorithm in dealing with large-scale problems is verified by testing the actual arithmetic cases in the planning area of the shared bicycle parking area layout between the North Second Ring Road and the Third Ring Road in Jinniu District, Chengdu City. Compared with the traditional method, the optimization method proposed in this paper can obtain a better scheme for the layout of shared bicycle parking area and delivery quantity, and reduce the investment cost of shared bicycle enterprises based on the travel demand of residents at each basic demand point. In future research, the layout of shared bicycle parking areas and delivery quantity under the conditions of joint operation of multiple shared bicycle enterprises can be further considered.

## Data Availability

The datasets generated and/or analyzed during the current study are not publicly available due [REASON WHY DATA ARE NOT PUBLIC] but are available from the corresponding author on reasonable request.
